# Relationship of Inflammatory Cytokines From M1-Type Microglia/Macrophages at the Injured Site and Lumbar Enlargement With Neuropathic Pain After Spinal Cord Injury in the CCL21 Knockout (*plt*) Mouse

**DOI:** 10.3389/fncel.2019.00525

**Published:** 2019-11-21

**Authors:** Kazuya Honjoh, Hideaki Nakajima, Takayuki Hirai, Shuji Watanabe, Akihiko Matsumine

**Affiliations:** Department of Orthopaedics and Rehabilitation Medicine, Faculty of Medical Sciences University of Fukui, Fukui, Japan

**Keywords:** CCL21, neuropathic pain, spinal cord injury, macrophage, microglia, M1/M2 phenotype, *plt* mouse

## Abstract

Spinal cord injury (SCI) causes loss of normal sensation and often leads to debilitating neuropathic pain (NeP). Chronic NeP develops at or below the SCI lesion in as many as 80% of patients with SCI and may be induced by modulators of neuronal excitability released from activated microglia and macrophages. In the inflammatory response after SCI, different microglia/macrophage populations that are classically activated (M1 phenotype) or alternatively activated (M2 phenotype) have become of great interest. Chemokines have also recently attracted attention in neuron-microglia communication. CCL21 is a chemokine that activates microglia in the central nervous system (CNS) and is expressed only in neurons with an insult or mechanical injury. In this study using an SCI model in mutant (*plt*) mice with deficient CCL21 expression, we assessed post-SCI NeP and expression of microglia/macrophages and inflammatory cytokines at the injured site and lumbar enlargement. SCI-induced hypersensitivities to mechanical and thermal stimulation were relieved in *plt* mice compared with those in wild-type (C57BL/6) mice, although there was no difference in motor function. Immunohistochemistry and flow cytometry analysis showed that the phenotype of microglia/macrophages was M1 type-dominant in both types of mice at the lesion site and lumbar enlargement. A decrease of M1-type microglia/macrophages was seen in *plt* mice compared with wild-type, while the number of M2-type microglia/macrophages did not differ between these mice. In immunoblot analysis, expression of M1-induced cytokines [tumor necrosis factor-α (TNF-α), interferon-γ (IFN-γ)] was decreased in *plt* mice, while that of M2-induced cytokines interleukin-4 (IL-4, IL-10) did not differ in the two types of mice. The results of this study indicate that suppression of expression of inflammatory cytokines by decreasing the number of M1-type microglia/macrophages at the injured site and lumbar enlargement is associated with provision of an environment for reduction of NeP. These findings may be useful for the design of new therapies to alleviate NeP after SCI.

## Introduction

The International Association for the Study of Pain defines neuropathic pain (NeP) as that associated with anatomical or functional abnormalities of the nervous system (Merskey and Bogduk, 1994). Spinal cord injury (SCI) results in loss of normal sensation and often causes debilitating NeP such as allodynia that is a persistent problem for many patients. Pain at the level of the spinal segment occurs in 37–50% of these patients, while 76–83% have pain below the lesion level (Ravenscroft et al., 2000; Turner et al., 2001; Siddall et al., 2003; Calmels et al., 2009; Jensen and Finnerup, 2014; Nagoshi et al., 2016). These symptoms are associated with significant impairment in health-related quality of life (Woolf and Mannion, 1999; Jensen et al., 2008; Doth et al., 2010; Finnerup et al., 2015; Inoue et al., 2017; Nakajima et al., 2019), but current medications are often ineffective for NeP after SCI. Therefore, greater attention to NeP is required since it is clinically important to lessen pain.

The underlying mechanisms of NeP after SCI are multifactorial and change with time, but spinal and supraspinal lesions are the main causes of NeP. Several studies of the pathomechanism of NeP after SCI have shown that monocytes, macrophages, and especially glial cells play important roles (Watanabe et al., 2015; Gwak et al., 2017; Chen et al., 2018). In particular, two subtypes of macrophages have become of great interest in SCI: classically activated macrophages (M1 phenotype) and alternatively activated macrophages (M2 phenotype; Gordon and Martinez, 2010; David and Kroner, 2011). The M1 phenotype is the product of exposure to T helper 1 (Th1) cytokines, such as interferon-γ (IFN-γ) and tumor necrosis factor-α (TNF-α). In contrast, the M2 phenotype is activated *via* T helper 2 (Th2) cytokines, such as interleukin (IL)-4 and IL-10 (Kigerl et al., 2009; Gordon and Martinez, 2010).

The M1 phenotype strongly expresses inflammatory cytokines that may be responsible for NeP, while the M2 phenotype has enhanced anti-inflammatory properties. Microglia can also be induced to M1 and M2 phenotypes under different conditions (David and Kroner, 2011), and activated microglia and macrophages cause allodynia after SCI at the injured site and at remote sites, such as in the brain and lumbar enlargement (Wu et al., 2014). We have shown that these M1-type cells are involved in post-SCI dorsal horn hyperexcitability and central NeP associated with pain-related substances (Matsuo et al., 2014; Watanabe et al., 2015).

**Figure 1:**
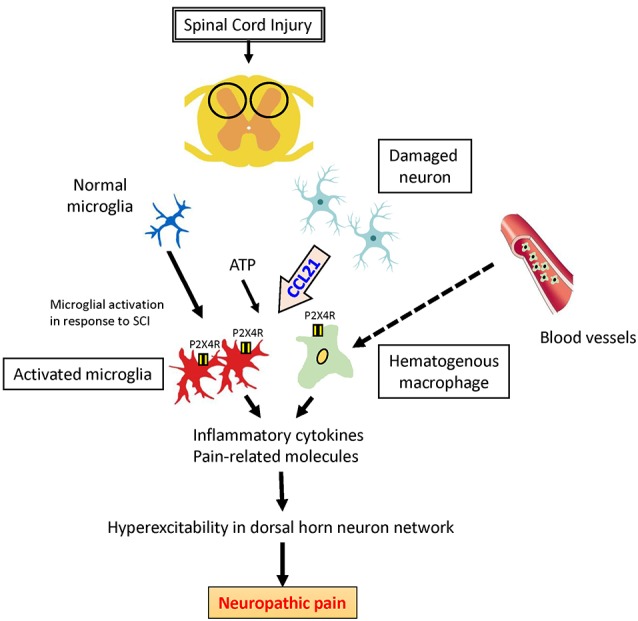
Potential role of CCL21 in neuropathic pain (NeP) after spinal cord injury (SCI). CCL21 secreted by damaged neurons is the trigger to upregulate P2X4 receptors activated by adenosine triphosphate (ATP) in activated microglia and hematogenous macrophages, which might lead to NeP.

There is increasing recognition of involvement of the chemokine CCL21 in initiation and maintenance of allodynia. In a peripheral nerve injury model, CCL21 is only expressed in damaged neurons and induces upregulation of the P2X4 receptor in microglia and macrophages. These P2X4 receptor-expressing cells are activated by adenosine triphosphate (ATP) and release pain-related factors such as brain-derived neurotrophic factors (BDNF) and/or inflammatory cytokines such as TNF-α, IFN-γ, and IL-6; and these events promote NeP after SCI (Figure 1; Biber et al., 2011; Tsuda et al., 2013). These findings indicate that CCL21 has a specific role in neuron-microglia/macrophage communication and is a potential drug target for prevention of NeP.

Several studies have analyzed the role of CCL21 in NeP in a peripheral nerve injury model, but little is known in SCI, including the effects on M1- and M2-type microglia/macrophage chemotaxis at the injured site and in remote regions. In this study, we assessed expression of inflammatory cytokines associated with M1- and M2-type activated microglia/macrophages at the injured site and lumbar enlargement after SCI in mutant (*plt*) mice with deficient CCL21 expression, in order to identify the role of CCL21 in NeP after SCI.

## Materials and Methods

### Experimental Animals

The study was conducted using C57BL/6 mice (age 10–12 weeks, male, *n* = 54) and *plt* (paucity of lymph node T cell) mice (age 10–12 weeks, male, *n* = 54; Mori et al., 2001; Nakano and Gunn, 2001) purchased from the Central Institute for Experimental Animals (Kawasaki, Japan). The *plt* mouse is a colony of DDD/1 inbred mice exhibiting greatly diminished T cell numbers in lymph nodes. This spontaneous deletion, designated *plt*, behaves as a single autosomal recessive allele and deletes a portion of chromosome 4, including both the *Ccl19* and *Ccl21a* loci. The Animal Ethics Review Committee of Fukui University approved the study protocol.

### Contusion SCI Model

After treatment with isoflurane [2% (v/v); Forane; Abbot, Tokyo, Japan] to induce deep anesthesia, the mouse underwent laminectomy at the T9–10 vertebral level to expose the spinal cord, with use of a surgical microscope (Vanox; Olympus Optical, Tokyo, Japan). An Infinite Horizon Impacter (Precision Systems and Instrumentation LLC, Fairfax, VA, USA) was used to produce a contusion SCI model, using an impact force of 60 kilodynes. For sham SCI, laminectomy only was performed at T9-T10, with no SCI. The wound and surrounding skin were sutured with 5–0 silk.

### Behavioral and Sensory Testing

The Basso Mouse Locomotor Scale (BMS) is used to evaluate locomotor function after thoracic spinal cord contusion or transection injury (Basso et al., 2006). It is scored from 0 (hind limb paralysis) to 9 (normal locomotion). BMS scores were recorded at days 4, 14, and 28 post-SCI for each hind limb, and averaged to give one value per mouse per test. Two independent examiners who were blinded to the experimental results tested the mice at days 1, 14, 21, and 28 post-SCI for mechanical allodynia and thermal sensitivity. Allodynia sensitivity was tested using a Dynamic Plantar Aesthesiometer (Ugo Basile, Comerio, Italy; Martucci et al., 2008). In this test, the withdrawal threshold (expressed in grams) is determined five times and the mean is reported. Thermal sensitivity at the plantar hindpaws was examined using a Plantar Test Apparatus (Ugo Basile; Hargreaves et al., 1988). In this test, the time between application of the thermal stimulus until hindpaw withdrawal (latency) is recorded (in seconds), as well as any other reaction to the stimulus (e.g., gazing at the affected paw, sniffing, licking, or attacking the stimulus). The latency was calculated using data from six tests after rejecting the longest and shortest latencies (Hoschouer et al., 2010).

### Immunohistochemistry

For immunohistochemical analysis, mice were deeply anesthetized and transcardially perfused at days 4, 14 and 28 post-SCI, and the obtained tissues were fixed with 4% paraformaldehyde in 0.1 M phosphate-buffered saline (PBS). The spinal cord and the lumbar enlargement at L3-L4 were dissected out carefully and kept in a similar fixative. After a few hours in the fixative solution, the tissue samples were immersed in a mixture of 10% sucrose/0.1 M PBS and maintained at 4°C for 24 h, and then in another solution of 20% sucrose/0.1 M PBS for another 24 h. The injured site and lumbar enlargement of the spinal cord were embedded in OCT (optimal cutting temperature) compound (Sakura Finetek, Torrance, CA, USA) and then cut into serial 20-μm axial or sagittal frozen sections using a cryostat. The cut sections were serially mounted on glass slides and fixed for 5 min with 2% paraformaldehyde in 0.1 M PBS, followed by rinsing in PBS and storage at −80°C.

Immunohistochemical staining was performed after permeabilization of the frozen sections with 0.1 M Tris-HCl buffer (with 0.3% Triton X-100, pH of 7.6). The sections were treated overnight with the following primary antibodies at 4°C, which were diluted with the Antibody Diluent with Background Reducing Components (Dako Cytomation, Carpinteria, CA, USA): mouse rat anti-CD11b monoclonal antibody (Abcam plc, ab1211, Cambridge, UK, diluted 1:200) for microglia/macrophages; rabbit anti iNOS (Proteintech, 18985-A-P, Chicago IL, USA, dilution 1:50) for M1-type macrophages; and rabbit anti-mannose receptor antibody (CD206; Abcam plc, ab64693, diluted 1:500). The sections were then incubated with Alexa Fluor-conjugated 488 or 568 secondary antibodies (dilution, 1:250, Molecular Probes, Eugene, OR, USA) for 1 h at room temperature. Finally, the sections were washed, wet-mounted, and examined by fluorescence microscopy (Olympus AX80, Olympus Optical, Tokyo) or a confocal laser scanning microscopy (TCS SP2, Leica Instruments, Nussloch, Germany), using an argon/helium-neon laser at 488 and 543 nm for fluorescence excitation.

### Semi-quantitative Analysis

Semi-quantitative analysis of the numbers of CD11b^+^/iNOS^+^ and CD11b^+^/CD206^+^ cells (merged cells; yellow) at days 4, 14, 28 post-SCI was performed in five axial sections selected randomly at about ±500 μm from the epicenter of the injured site and at the L3-L4 level for the lumbar enlargement. High magnification (×200) photomicrographs (TCS SP2; Leica Microsystems) of superficial laminae I-III on one side of the spinal dorsal horn were analyzed using grain counting with the light intensity automatically set by the color image analysis software (MacSCOPE; Mitani; Hansen et al., 2013, 2016; Watanabe et al., 2015). The light intensity and threshold values were maintained at constant levels when collecting digitized images in all analysis.

### Immunoblot Analysis

For immunoblot analysis, the injured site (tissue harvested from 2.5 mm on either side of the injured site) and lumbar enlargement (between L3 and L4) were carefully dissected *en bloc* from the area (5 mm; *n* = 3 mice at each time point) and stored at −80°C. Sections were centrifuged at 15,000× *g* for 30 s (BioMasher Rapid Homogenization Kit, Funakoshi, Tokyo) and then solubilized in RIPA lysis buffer 1× (Santa Cruz Biotechnology, Santa Cruz, CA, USA), homogenized and stored at −80°C. Protein concentrations in tissue samples were determined by Lowry protein assay (DC Protein Assay Kit; Bio-Rad Laboratories, Hercules, CA, USA). Protein mixtures were mixed with Laemmli sodium dodecylsulfate buffer and boiled prior to immunoblot analysis. The total protein (20 μg/lane) was separated on 12.5% SDS-PAGE and transferred onto a polyvinylidene difluoride membrane (PE Applied Biosystems, Foster City, CA, USA) for 70 min. The membranes were washed twice in PBS solution containing 0.05% Tween 20, then blocked with a mixture of 5% skimmed milk in PBS for 1 h at room temperature, and finally incubated overnight at 4°C with an antibody (all Abcam plc) against the following proteins: TNF-α (ab6671, dilution 1:500), IFN-γ (ab133566, dilution 1:1,000), IL-4 (ab11524, dilution 1:500), and IL-10 (ab33471, dilution 1:500) in blocking solution. After washing three times in 0.1 M PBS, the membranes were immersed in medium from an ECL Advance Western Blot Detection kit (GE Healthcare, Little Chalfont, UK) for 1 min and analyzed by imaging (Image Quant LAS 4000, GE Healthcare Life Science, Piscataway, NJ, USA). Each band intensity was quantified using Image Quant TL software (GE Healthcare Life Science) and expressed relative to the intensity of the band for β-actin. Kaleidoscope Prestained Standards (Bio-Rad Laboratories) were used as molecular weight controls.

### Flow Cytometric Analysis

Flow cytometric analysis was conducted using tissues harvested from 2.5 mm on either side of the injured site and between L3 and L4 of the spinal cord at days 4 and 14 post-SCI, as described previously (Watanabe et al., 2015). Hematogenous macrophages/activated microglia were detected as CD45^+^/CD11b^+^/GR-1^−^ cells (Saiwai et al., 2010). For intracellular staining (Stirling and Yong, 2008), the harvested cells were resuspended in fixation buffer and then treated with permeabilization buffer (Santa Cruz Biotechnology). They were then resuspended in ice-cold PBS and incubated for 1 h with one of the following antibodies: BV510 rat anti-CD11b (BD Horizon, 562950, Piscataway, NJ, USA, dilution 1:1,000), FITC rat anti-CD45 (Abcam plc, ab25670, dilution 1:1,000), and PerCP/Cy5.5 rat anti-Ly-6G/Ly-6C, equivalent to Gr-1 (BioLegend, 108427, San Diego, CA, USA, dilution 0.25 μg for 10^6^ cells), rabbit anti-iNOS antibody-primary antibody (Abcam plc, ab15323, dilution 1:500), Alexafluor 647 equivalent to EPR25A secondary antibody (Abcam plc, ab199093, dilution 1:2,000) for M1 or EPR6828(B) rabbit anti-mannose receptor antibody equivalent to CD206 (Abcam plc, ab195192, dilution 1:50) for M2. Flow cytometry (FACSCanto™ II; BD Biosciences) was then performed with forward scattering to eliminate cellular debris. CD45^+^/CD11b^+^/GR-1^−^/iNOS^+^ cells and CD45^+^/CD11b^+^/GR-1^−^/CD206^+^ cells were detected as hematogenous M1- and M2-type macrophages/activated microglia, respectively.

### Statistical Analysis

All values are expressed as mean ± standard deviation (SD). Differences between groups were examined for statistical significance using one-way factorial analysis of variance (ANOVA). A *p* < 0.05 denoted the presence of significant difference with Tukey’s *post hoc* analysis. The above tests were conducted using SPSS software (version 24.0, SPSS, Chicago, IL, USA).

## Results

### Locomotor Function, Mechanical Allodynia and Thermal Hyperalgesia After SCI

**Figure 2:**
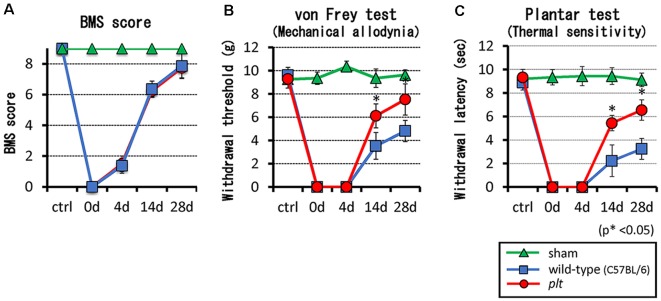
**(A)** There was no difference in Basso Mouse Scale (BMS) scores between *plt* and wild-type mice. **(B,C)** SCI-induced hypersensitivities to mechanical and thermal stimulation were reduced in *plt* mice from day 14 post-SCI (*n* = 10 for each time point). Data are shown as mean ± standard deviation (SD).

There was no difference in BMS locomotor scores between wild-type and *plt* mice after contusive SCI (Figure 2A). Hind paw mechanical and thermal sensitivity tests were performed from week 2 post-SCI, which was when all mice were able to place the plantar surface of the hind paws and support weight. The mechanical response thresholds at 2 and 4 weeks post-SCI were 3.53 g and 4.82 g, respectively, in wild-type mice, and 6.12 g and 7.53 g, respectively, in *plt* mice, showing a significant decrease in mechanical hypersensitivity in *plt* mice (Figure 2B). In the thermal sensitivity test, the withdrawal latency of wild-type mice gradually increased from 2.23 s at 2 weeks to 3.25 s at 4 weeks post-SCI. In contrast, a significant and marked improvement in thermal sensitivity was noted in *plt* mice from 2 weeks post-SCI, with withdrawal latency times of 5.44 s at 2 weeks and 6.55 s at 4 weeks post-SCI (Figure 2C).

### Expression of M1- and M2-Type Microglia/Macrophages

**Figure 3:**
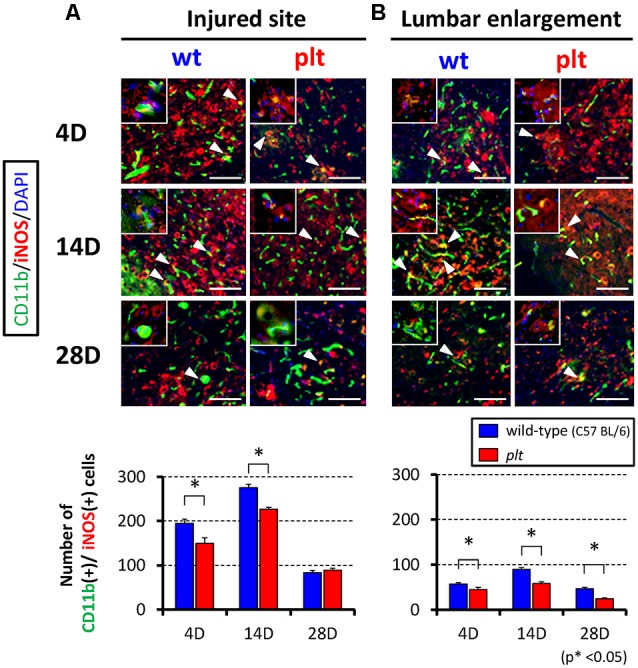
Immunofluorescent staining showing expression of M1-type microglia/macrophages at the injured site and lumbar enlargement after SCI using CD11b (green), iNOS (red) and DAPI (blue). Suppression of increases in CD11b^+^/iNOS^+^ cells (M1-type microglia/macrophages; merged cells; yellow) in *plt* mice at the injured site **(A)** and lumbar enlargement **(B)**. Scale bars = 50 μm. The upper left panels in each image were high-power photomicrograph of the merged cells. The number of CD11b^+^/iNOS^+^ cells was significantly decreased at days 4 and 14 post-SCI at the injured site **(A)**, and at days 4, 14 and 28 post-SCI at the lumbar enlargement (*n* = 4 at each time point). Data are shown as mean ± SD. *p < 0.05.

**Figure 4:**
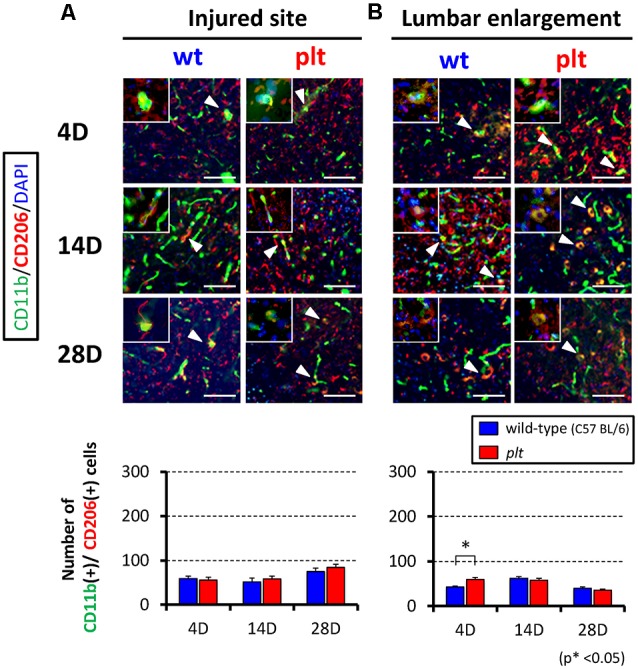
Immunofluorescent staining showing expression of M2-type microglia/macrophages at the injured site and lumbar enlargement after SCI using CD11b (green), CD206 (red) and DAPI (blue). Expression of CD11b^+^/CD206^+^ cells (M2-type microglia/macrophages; merged cells; yellow) did not differ between wild-type and *plt* mice at the injured site **(A)** and lumbar enlargement **(B)**. Scale bars = 50 μm. The upper left panels in each image were high-power photomicrograph of the merged cells. The number of CD11b^+^/CD206^+^ cells did not differ significantly from day 4 to day 28 post-SCI at the injured site **(A)** and lumbar enlargement (**B**; *n* = 4 for each time point). Data are shown as mean ± SD. *p < 0.05.

Expression of M1- and M2-type microglia/macrophages at the injured site and lumbar enlargement after SCI was examined by immunohistochemistry. The number of CD11b^+^/iNOS^+^ cells (M1 type) at the injured site peaked at 14 days after SCI in wild-type mice, and some of the CD11b-positive cells seemed to form clusters. There was significant suppression of the increase in these cells in *plt* mice at 4 and 14 days after SCI (Figure 3A). In the lumbar enlargement, the number of CD11b^+^/iNOS^+^ cells tended to increase from 4 days to 14 days after SCI in both types of mice, but there was significant suppression of the increase in these cells in *plt* mice at 4, 14, and 28 days after SCI, compared with the number in wild-type mice (Figure 3B). The number of CD11b^+^/CD206^+^ cells (M2 type) after SCI was smaller than that of CDD11b^+^/iNOS^+^ cells (M1 type) and did not differ at the injured site in wild-type and *plt* mice (Figure 4A). At the lumbar enlargement, there was a significantly larger number of these cells at 4 days after injury, but no differences at 14 and 28 days after injury (Figure 4B).

### Flow Cytometry for M1/M2 Hematogenous Macrophages and Activated Microglia

**Figure 5:**
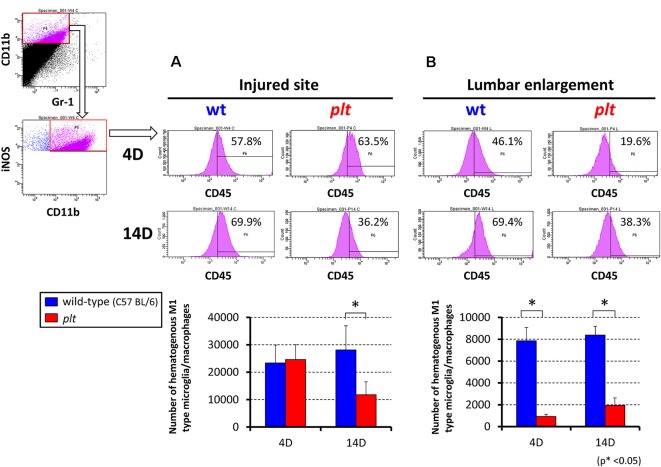
Semi-quantitative flow cytometric analysis of M1-type activated microglia/macrophages at the injured site and lumbar enlargement in wild-type and *plt* mice. Flow cytometry of CD11b^+^/CD45^+^/Gr-1^−^ cells showed a significant decrease in iNOS-positive hematogenous M1 type microglia/macrophages at day 14 in the injured site** (A)** and at day 4 and 14 in the lumbar enlargement **(B)** after injury in *plt* mice compared with those in wild-type mice (*n* = 3 each). Data are shown as mean ± SD. *p < 0.05.

**Figure 6:**
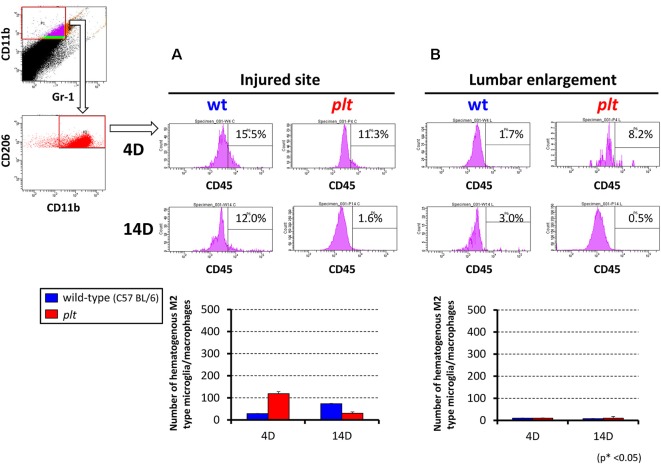
Semi-quantitative flow cytometric analysis of M2-type activated microglia/macrophages at the injured site and lumbar enlargement in wild-type and *plt* mice. The number of M2-type cells after SCI was <5% in sorted CD11b^+^/CD45^+^/Gr-1^−^ hematogenous cells at the injured site **(A)** and lumbar enlargement **(B)**, with no difference at days 4 and 14 after injury between wild-type and *plt* mice (*n* = 3 each). Data are shown as mean ± SD.

Flow cytometry was used to examine the number of M1- and M2-type hematogenous macrophages/activated microglia in 100,000 sorted cells at the injured site and lumbar enlargement. There was a decrease in M1-type cells (CD11b^+^/CD45^+^/Gr-1^−^/iNOS^−^) at 14 days after SCI at the injured site in *plt* mice (28,162 ± 8,683 in wild-type vs. 11,815 ± 6,529 in* plt* mice), but no difference at 4 days (23,357 ± 6,522 vs. 24,622 ± 5,430; Figure 5A). In the lumbar enlargement, the number of M1-type cells was significantly lower in *plt* mice than in wild-type mice at 4 days (7,860 ± 1,210 vs. 1,065 ± 202) and 14 days (8,382 ± 810 vs. 1,941 ± 684) after SCI (Figure 5B). There were few M2-type cells (CD11b^+^/CD45^+^/Gr-1^−^/CD206^+^) after SCI among sorted cells, with no difference between wild-type and *plt* mice at the injured site and lumbar enlargement (Figure 6).

### Expression of M1- and M2-Induced Inflammatory Cytokines

**Figure 7:**
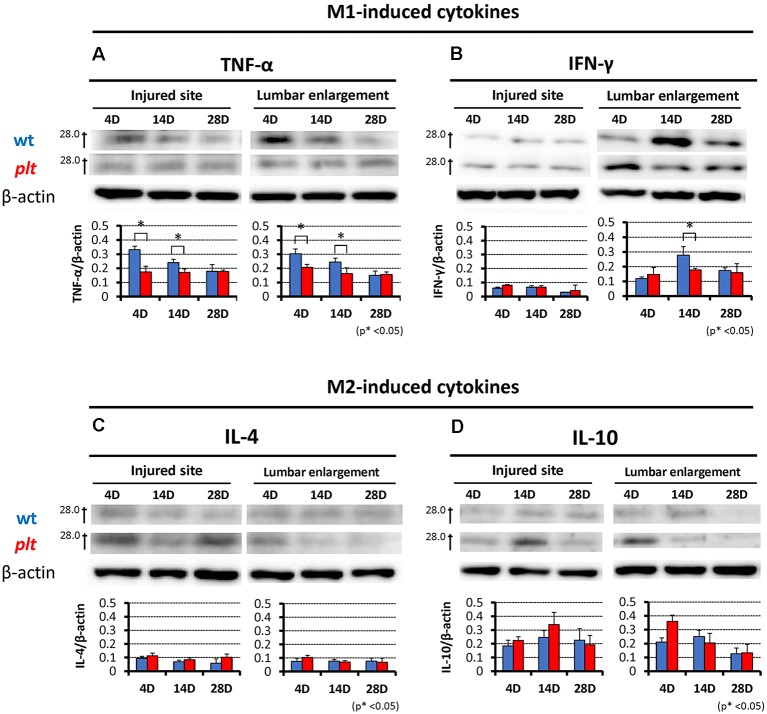
Western blotting showing expression of M1- and M2-induced cytokines at the injured site and lumbar enlargement after SCI in wild-type and *plt* mice. **(A)** Expression levels of tumor necrosis factor-α (TNF-α) were significantly decreased from day 4 to day 14 after injury at the injured site and lumbar enlargement in *plt* mice compared with those in wild-type mice (*n* = 3 each).** (B)** The expression level of interferon-γ (IFN-γ) did not differ at the injured site from day 4 to day 28 after injury, but was significantly decreased at day 14 after injury at the lumbar enlargement in *plt* mice compared with wild-type mice (*n* = 3 each). Expression levels of interleukin-4 (IL-4; **C**) and IL-10 **(D)** did not differ between wild-type and *plt* mice at the injured site and lumbar enlargement from days 4 to 28 after injury (*n* = 3 each). Data are shown as mean ± SD. *p < 0.05.

Western blot analysis showed total protein level of M1-induced pro-inflammatory cytokines (TNF-α, IFN-γ) and M2-induced anti-inflammatory cytokines (IL-4, IL-10) at the injured site and lumbar enlargement after SCI. In *plt* mice, expression of TNF-α at both the injured site and lumbar enlargement was significantly reduced at 4 and 14 days after SCI, compared with wild-type mice. At 28 days after SCI, there was almost no difference in the expression of TNF-α between the two types of mice (Figure 7A). Expression of IFN-γ did not differ at the injured site, but decreased expression was seen at 14 days after injury at the lumbar enlargement in *plt* mice (Figure 7B). Expression of IL-4 and IL-10 did not differ, although these levels were slightly increased in *plt* mice at both the injured site and lumbar enlargement (Figures 7C,D).

## Discussion

The main finding in this study was that NeP after SCI was reduced in *plt* mice, which have deficient CCL21 expression, due to decreased infiltration of M1-type hematogenous macrophages/activated microglia and reduced expression of pro-inflammatory cytokines without affecting M2-type chemotaxis at the injured site and lumbar enlargement. This suggests that CCL21 could be a potential drug target for prevention of NeP in response to SCI.

CCL21 is a chemokine that is present in humans and has an important role in mobilizing normal immune cells in response to tumor cells metastasizing to lymph nodes, *via* activation of a G protein-coupled receptor, CCR7 (Love et al., 2012). Following storage in large dense core vesicles, neuronal CCL21 is transported along axons in an anterograde direction to presynaptic terminals, indicating the function of CCL21 in neuron-microglia signaling (de Jong et al., 2008). The purinergic P2X receptors, of which seven subtypes (P2X1R-P2X7R) have been identified, are a family of ligand-gated cation channels. Activated microglia express several subtypes of these receptors, which play a key role in establishing and maintaining NeP states (Tsuda et al., 2013). In NeP after SCI, CCL21 is secreted from damaged neurons and induces P2X4 receptor upregulation in microglia activated by extracellular ATP released from dying cells. Release of pain-related factors from activated microglia with P2X4 receptor expression induces hyperexcitability in the dorsal horn pain network, which may be responsible for NeP (Biber et al., 2011; Tsuda et al., 2013).

Macrophages in an injured spinal cord have dynamic phenotypes and functions that can change based on the spinal lesion microenvironment (Stout and Suttles, 2004; Menzies et al., 2010). CCL21 and its receptor CCR7 are widely expressed in T cells, dendritic cells, fibroblasts, smooth muscle, and intravascular cells, and CCL21 contributes to inflammation and remodeling of the extracellular matrix (Jiang et al., 2015). CCR7 has an important role to migrate dendritic cells and/or T cells from brain parenchyma to deep cervical lymph nodes for immune response; CCR7 deficient inflammatory cells are retained in the central nervous system (CNS) and exacerbate CNS autoimmune diseases, including multiple sclerosis (Kivisäkk et al., 2004; Clarkson et al., 2017). However, CCR7 is absent in the spinal cord and CCL21 has not been detected in healthy neurons, glial cells and other non-neuronal cells in the CNS (Biber and Boddeke, 2014). Recent reports indicate that CCR7 is specifically expressed in M1 cells and triggers their migration. Expression of CCR7 is not found in M0 microglia/macrophages before differentiation into M1/M2 and M2 phenotypes (Bellora et al., 2010). The study using cultured human macrophage demonstrated that CCR7 was expressed exclusively on the cell surface of M1 but in the cytosol of M2 macrophages and that both CCL19 and CCL21 activated MEK1-ERK1/2 and PI3K-AKT pathways in M1 but not in M2 macrophages (Xuan et al., 2015). In the other study, very few T cells were found in the spinal cord after spinal nerve injury with no differences between wild-type controls and *plt* mice (Biber et al., 2011). Early and long-lasting microglia reactivity in the spinal cord after nerve injury was found, but there was no lymphocyte infiltration (Gattlen et al., 2016). Microglia activation in the spinal cord involves both hypertrophy and hyperplasia. This process progresses through a hypertrophic morphology, with thickened and retracted processes, and an increase in cell number (Smith, 2010). Numerous factors, such as cytokines, chemokines and ATP, can induce morphological microglia activity. These factors are expressed and probably released in the spinal cord after spinal nerve injury. This may account for the morphological activation of microglia. However, microglia morphology is not a sufficient readout of their function, since morphologically activated microglia do not cause development of tactile allodynia in the absence of CCL21 and subsequent P2X4 receptor upregulation (Biber et al., 2011). These findings suggest a specialized role of CCL21 in the recruitment of M1 macrophages, but not of other inflammatory cells, in the injured spinal cord. However, it should be noted that experiments isolating cells of a particular day need to be provided to analyze M1 and/or M2 chemotaxis towards CCL21. CCL21 has been shown to be involved in below-level allodynia after SCI by causing M1 chemotaxis (Xuan et al., 2015). We previously showed that M1-type active microglia and macrophages that are increased after SCI express pain-related substance and contribute to NeP, and that a change in polarity of microglia/macrophages (M1/M2 phenotype) has roles as a trigger for worsening neuroinflammation (Matsuo et al., 2014; Watanabe et al., 2015). In the current study, we found a decrease in the number of M1-type cells in *plt* mice after SCI at the injured site and lumbar enlargement, with no difference in the number of M2-type cells. There was no difference in motor function between wild-type and *plt* mice, but the decrease of M1-type macrophage infiltration may have beneficial effects on motor function in cases with more severe spinal cord damage. This is a matter for a future study.

With regard to below-level NeP, expression of TNF-α, IL-1β and IL-6 in the lumbar enlargement after midthoracic SCI is related to the severity of below-level allodynia. Early increases in TNF-α may promote induction of below-level allodynia (Detloff et al., 2008), and M1-type macrophages themselves produce high levels of inflammatory molecules such as TNF-α, IFN-γ and iNOS (Zhou et al., 2014). TNF-α and IFN-γ produced by M1-type microglia/macrophages spread to the lumbar enlargement following SCI, leading to activation of resident microglia and infiltration of inflammatory cells due to the hyperpermeability of the blood spinal cord barrier (Peng et al., 2006; Liu et al., 2007). Moreover, extracellular ATP stimulated TNF-α/IFN-γ induced cell communication in microglia, which might serve to amplify inflammatory signals (Davalos et al., 2005; Sáez et al., 2013). CCL21 has been detected at the site of injury and also at remote sites in SCI, including in brain regions and the lumbar enlargement, due to transport of CCL21 *via* axons. Distal release of CCL21 triggers microglial activation at the distant site, which is essential for development of central NeP and below-level pain (Wu et al., 2014). In this study, expression levels of TNF-α and IFN-γ in *plt* mice were decreased at the lumbar enlargement from day 4 to day 14 after SCI, which may be associated with the decreased M1-type microglia/macrophage chemotaxis to the lumbar enlargement.

These results indicate that prevention of neuronal CCL21 expression at an injured site could serve as preventive therapy for NeP at a site distant from the lesion. In a peripheral nerve injury model, intrathecal injection of CCL21 in *plt* mice induces long-lasting formation of tactile allodynia, and CCL21-blocking antibody treatment reduced development of tactile allodynia in wild-type mice. These results indicate that prevention of CCL21 expression after SCI could reduce NeP. There are various cytokines associated with NeP after SCI, and it is important to avoid adverse effects by blocking cytokines with drugs. CCL21 only induced M1-type microglia/macrophage chemotaxis without affecting M2-type chemotaxis in this study; therefore, prevention of overexpression of CCL21 may not induce adverse effects. A phase I clinical trial of antagonists of the P2X4 receptor (CCL21 up-regulates this receptor in microglia/macrophages) in Japan has confirmed safety and delivery to the CNS. However, possible upstream therapy should be considered, along with P2X4 receptor antagonists. CCL21-blocking antibody treatment may be more effective in an earlier phase after injury because expression of CCL21 reached a peak early after SCI and diminished thereafter. In a peripheral nerve injury model, there was no increase in pain for 10 days after withdrawal of a CCL21-blocking antibody (Biber et al., 2011). CCL21 is an initiation factor for NeP, and it is important to inhibit the inflammatory reaction in an earlier phase to prevent induction of chronic pain, whereas the influence of a CCL21 blocker in the chronic phase may be limited.

## Conclusion

We show that NeP after SCI was reduced in *plt* mice with deficient CCL21 expression due to decreased infiltration of M1-type hematogenous macrophages/activated microglia and reduced expression of pro-inflammatory cytokines without affecting M2-type chemotaxis up to 28 days after SCI at the injured site and lumbar enlargement. These results suggest that CCL21 is a key cytokine for NeP after SCI. Our findings are potentially useful for design of new therapies to alleviate NeP after SCI.

## Data Availability Statement

The datasets generated for this study are available on request to the corresponding author.

## Ethics Statement

The animal study was reviewed and approved by the Animal Ethics Review Committee of Fukui University.

## Author Contributions

KH and HN designed various aspects of the study. KH, HN and AM wrote the final manuscript text. HN and SW prepared Figures 1, 2. KH and SW prepared Figures 3, 4, 7. KH and TH prepared Figures 5, 6. All authors reviewed the manuscript.

## Conflict of Interest

The authors declare that the research was conducted in the absence of any commercial or financial relationships that could be construed as a potential conflict of interest.
